# Genetic markers and predictive model for individual differences in countermovement jump enhancement after resistance training

**DOI:** 10.5114/biolsport.2024.136088

**Published:** 2024-04-09

**Authors:** Tao Mei, Xiaoxia Li, Yanchun Li, Xiaolin Yang, Liang Li, Zihong He

**Affiliations:** 1 China Institute of Sport and Health Science, Beijing Sport University, Beijing, China; 2 Department of Teaching Affairs, Shandong Sport University, Jinan, China; 3 Sultan Idris Education University, Tanjung Malin, Malaysia; 4 Biological Science Research Center, China Institute of Sport Science, Beijing, China

**Keywords:** Genome-wide association study, Resistance training, Countermovement jump, Personalized exercise, Predictive model

## Abstract

This study aims to utilize Genome-Wide Association Analysis (GWAS) to identify genetic markers associated with enhanced power resulting from resistance training. Additionally, we analyze the potential biological effects of these markers and establish a predictive model for training outcomes. 193 Han Chinese adults (age: 20 ± 1 years) underwent resistance training involving squats and bench presses at 70% 1RM, twice weekly, 5 sets × 10 repetitions, for 12 weeks. Whole-genome genotyping was conducted, and participants’ countermovement jump (CMJ) height, lower limb muscle strength, and body muscle mass were assessed. CMJ height change was used to assess changes in power and subjected to Genome-Wide Association Analysis (GWAS) against genotypes. Employing Polygenic Score (PGS) calculations and stepwise linear regression, a predictive model for training effects was constructed. The results revealed a significant increase in CMJ height among participants following the resistance training intervention (Δ% = 16.53%, p < 0.01), with individual differences ranging from -35.90% to 125.71%. 38 lead SNPs, including PCTP rs9907859 (p < 1 × 10^−8^), showed significant associations with the percentage change in CMJ height after training (p < 1 × 10^−5^). The explanatory power of the predictive model for training outcomes, established using PGS and phenotypic indicators, was 62.6%, comprising 13.0% from PGS and 49.6% from phenotypic indicators. SNPs associated with power resistance training were found to participate in the biological processes of musculoskeletal movement and the Striated muscle contraction pathway. These findings indicate that individual differences in the training effect of CMJ exist after resistance training, partially explained by genetic markers and phenotypic indicators (62.6%).

## INTRODUCTION

In resistance training effects, researchers often concentrate on the average changes within a group, paying less attention to individual differences. Even with rigorously designed experiments and the use of homogeneous samples, results can still vary significantly due to individual differences. Assessing resistance training effects, studies have discovered significant individual variations in muscle strength improvements during resistance training, ranging from -11% to 30% in muscle size, -8% to 60% in 1RM, and -32% to 149% in isometric muscle strength [1, 2]. When reporting mean values, individual response differences are often masked. Some researchers suggest that sports science should prioritize individual data over average data. Focusing on individual differences is more meaningful for tailoring personalized training and enhancing training efficiency.

Individual differences in training effects are influenced by multiple factors, where genetics might be a key intrinsic factor. For instance, a meta-analysis found diverse responses to aerobic training among carriers of different genotypes [3]. Certain carriers of *PPARGC1A* and *PPARD* genotypes were categorized as non-responders, while others were considered negative or optimal responders to aerobic training [3]. In resistance training, approximately 2.1% of resistance training effects (elbow flexor isometric) can be attributed to the *ACTN3* gene [4]. The *IL15RA* genes rs3136617 and rs2296135 respectively explain 3.5% and 7.1% of the regression model variance in lean body mass training effects [5]. Moreover, an intronic marker of the *GLI3* gene can distinguish muscle fiber hypertrophy caused by resistance training in young males [6]. However, it’s crucial to note that these percentages might require further confirmation and replication, as such large variations in a complex trait from individual common variants might need validation through additional research.

Countermovement Jump (CMJ) reflects the power of lower limb muscle groups or neuromuscular status, a pivotal indicator for lower limb power [7]. Although specific exercises can improve power more effectively [8], conventional resistance training is equally effective in enhancing muscle power [9]. This study focuses on the genetic factors involved in improving CMJ through resistance training. The heritability of power measured by CMJ is approximately 0.55 [10], suggesting that individual differences in vertical jump training effects after resistance training may be genetically influenced. Additionally, phenotypic indicators such as age, gender, and strength levels may also influence the training effects of power. However, there is currently no reported research, especially at the whole-genome level analysis.

Therefore, this study aims to first explore individual differences in CMJ changes after 12 weeks of regular resistance training intervention. Secondly, through Genome-Wide Association Analysis (GWAS), it aims to screen SNPs related to CMJ changes after resistance training. Utilizing multi-gene scoring and predictive model construction, the study intends to analyze the explanatory power of SNPs on training effects. Finally, we will conduct bioinformatics analysis on the lead SNPs selected by GWAS to understand potential mechanisms influencing CMJ changes after resistance training. This endeavor aims to provide operational pathways for developing precise fitness guidance and lay the foundation for further exploration of the biological mechanisms of SNPs.

## MATERIALS AND METHODS

### Participants

Participants were selected based on the following inclusion criteria: (1) Participants had no prior experience with resistance training and were classified as non-regular exercisers using the Global Physical Activity Questionnaire (GPAQ); (2) Participants showed no risk of resistance training-related injuries as determined by the Physical Activity Readiness Questionnaire (PAR-Q); (3) Participants had no adverse dietary habits and maintained a regular diet during the intervention period, as determined by the Chinese Residents’ Nutrition and Health Survey Food Frequency Questionnaire; and (4) were of Han Chinese ethnicity in China. A total of 193 participants (95 males and 98 females, age: 20 ± 1 years, height: 172.5 ± 8.7 cm, body mass: 65.6 ± 13.0 kg) were included in the study. The study was conducted in accordance with the Declaration of Helsinki and approved by the Sports Science Experiment Ethics Committee.

### Resistance Training Program

The resistance training protocol employed in this study was based on a traditional resistance training program [11]. After a proper warmup, the participants initiated resistance training exercises, which consisted of squats and bench presses performed at 70% of their one-repetition maximum (1RM). Each participant completed 5 sets of 10 repetitions, with a 2-minute rest interval between sets. Participants were instructed to perform resistance training using a moderate or slower tempo. The training sessions were held twice a week for a duration of 12 weeks. At the end of every 4-week period,a one-repetition maximum (1RM) test was conducted to adjust the training loads based on the participants’ strength improvements. Throughout the intervention, close monitoring of the participants’ training loads was maintained, ensuring adherence to the prescribed training volume and standardized movement patterns. In cases where participants were unable to perform the exercises independently, minimal assistance was provided to ensure a consistent training load stimulus and maintain the same training content.

### Phenotypic Indicators Test

The tests encompass CMJ, 1RM, body composition, lower limb muscle thickness. The percentage change in CMJ height (Δ%) before and after the intervention is employed to evaluate the enhancement of power following strength training. Additionally, 1RM is utilized to determine training loads, whereas body composition and lower limb muscle thickness are considered as dependent variables when constructing predictive models.

### CMJ Testing

The measurement of lower limb power involved conducting the CMJ test. The test procedure was as follows: Participants commenced with warm-up activities to prepare and familiarize themselves with the test movements. Participants positioned themselves with hands on their hips on a force platform (Kistler, Switzerland). They rapidly squatted to a knee flexion angle of 90° and executed a quick jump. To minimize the influence of trunk movement on the test results, participants were instructed to maintain an upright position as much as possible during the airborne phase. Prior to the formal testing, a warm-up consisting of vertical jumps was performed. During the actual test, participants were asked to complete three consecutive CMJ tests, with a 15-second recovery period between each test. Participants remained standing during the recovery interval. The highest jump height recorded among the three tests was utilized for analysis. CMJ data were collected using Bioware software and were quantified using Kistler’s Measurement, Analysis, and Reporting Software (MARS).

### One Repetition Maximum

Lower limb strength was assessed by determining the one repetition maximum (1RM) weight for the squat exercise. Participants begin with a warm-up activity, which involves performing back squats/ bench presses at 40% of their subjective 1RM perception. After warming up, the weight is increased by 15–20 kg on top of the warm-up load, and they complete 3–5 back squats/bench presses. A rest period of 2–4 minutes follows, after which the weight is increased again by 15–20 kg (for back squats) or 5–10 kg (for bench presses), and they complete 2–3 back squats/bench presses. A further rest period of 2–4 minutes is taken, and the previous step is repeated. If the participant successfully completes the lift, continue to increase the load; if they fail, reduce the load by 5–10 kg (for back squats) or 2.5–5 kg (for bench presses) until they can complete 1RM with proper technique. The determination should be made within 5 attempts for both back squats and bench presses 1RM.

### Body Composition

Body composition was assessed using a GE lunar iDXA dual-energy X-ray absorptiometry (DXA) scanner (General Electric, USA). Before the test, participants were ensured to have not undergone a barium meal examination, received radioactive isotope injections, or took contrast agents orally or intravenously for CT and MRI scans within the past 7 days. Participants were needed to fast, remove any clothing that could affect the test results, and lie flat on the scanner table. EnCORE software was used to input participant information and set up the scanning parameters. The scanner performed a sequential scan from head to feet to obtain body composition data.

### Lower Limb Muscle Thickness

The GE portable color Doppler ultrasound diagnostic system LOGIQ e (General Electric, USA) was used to measure the rectus femoris and rectus femoris-vastus intermedius thickness. Prior to the measurements, the instrument was calibrated, and subject information was entered. The subjects were positioned in a supine position with their legs relaxed and positioned shoulder-width apart. A 12 MHz linear array probe was used to detect the midpoint between the anterior superior iliac spine and the superior border of the patella, and muscle thickness was obtained. Muscle thickness was measured on both sides, and three measurements were taken on each side to obtain the average value.

### Analysis of whole genome genetic polymorphisms

DNA samples that passed the quality control were subjected to wholegenome genotyping using the Infinium chip (Chip Type: CGA-24v1-0, Illumina Inc.). Before genotype imputation, genotypes underwent quality control following previously established methods [12, 13]. Imputation of genotype data was carried out using Eagle/Minimac4 with default parameters (chunk size of 10 Mb and step size of 3 Mb) against the 1000 Genomes project Phase3 v5 reference haplotypes. After imputation, quality control of the chip data was conducted using PLINK 1.9 software, following quality control criteria [14]: SNPs with a minor allele frequency (MAF) less than 5% (MAF < 0.05) were excluded, as well as those not conforming to Hardy-Weinberg equilibrium (HWE) (p < 1 × 10^−5^), SNPs with more than 10% missing genotypes, and individuals with a genotype missing rate exceeding 10%. After quality control, a total of 4,110,727 SNP loci were retained for subsequent GWAS analysis.

### Data Analysis

Data entry, organization, and statistical analysis were performed using Excel 2016 and SPSS 19.0. Descriptive statistics are presented as the mean ± standard deviation (mean ± SD). The normality of the data was assessed using the Kolmogorov-Smirnov test. The improvement in power training was represented by the percentage change in CMJ height before and after the intervention (Δ%). The overall evaluation of training effects was conducted using paired sample t tests, with a significance level of p < 0.05. Before GWAS analysis, population stratification was assessed using PCA. The PLINK 1.9 software was used for whole-genome association analysis, considering CMJ height baseline, sex, age, and the first five principal components from PCA as covariates. Genome-wide significance was set at the standard GWAS threshold of P < 5 × 10^−8^ and suggestive significance was set at p < 1 × 10^−5^ [15]. Lead SNPs (maximum p value of lead SNPs < 1 × 10^−5^) were identified using FUMA, and the corresponding mapping genes for these SNPs were analyzed. Polygenic scores (PGS) were calculated based on the lead SNPs, and the average PGS was calculated as per the following formula [16]:

$$\text{PGS}=\sum_{i}\frac{SNP_{i}\times beta_{i}}{n}$$

where SNP represents the variant allele (0, 1, or 2), beta signifies the effect size, and n denotes the number of SNPs. To evaluate the explanatory power of PGS and various phenotype indicators (height, body mass, age, sex, CMJ height, lower limb muscle strength, lower limb muscle mass, and lower limb muscle thickness) on the percentage change in CMJ height, stepwise regression analysis was performed. The percentage change in CMJ height served as the dependent variable (y), while PGS and phenotype indicators were used as independent variables (x), with gender coded as 0 for females and 1 for males. GO and pathway analysis was performed using Metascape, DAVID, KEGG, REACTOME, and Wikipathways.

## RESULTS

**FIG. 1:**
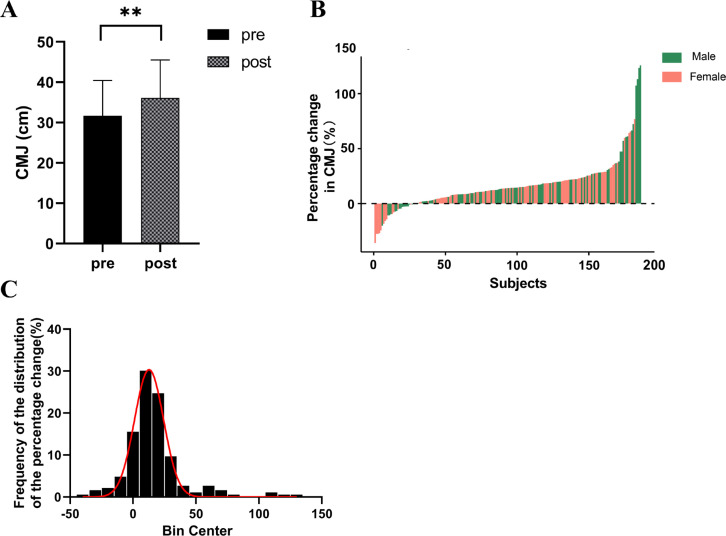
Individual Differences in Training Effects on Power Following Strength Training. A. CMJ height significantly increased after strength training intervention; B. Individual differences exist in the percentage change of CMJ height after strength training intervention; C: Distribution of CMJ height changes.

A significant enhancement in CMJ height was observed among the participants (ΔCMJ = 16.53%, p < 0.001) (Figure 1A). The magnitude of change varied considerably among individuals, ranging from -35.90% to 125.71%, with males showing changes between -20.14% to 125.71% and females between -35.90% to 76.85% (Figure 1B). Among the participants, 15.59% experienced a decrease or no change (ΔCMJ ≤ 0%), 63.98% witnessed an increase between 0% and 25% (0% < ΔCMJ ≤ 25%), 13.44% observed an increase between 25% and 50% (25% < ΔCMJ ≤ 50%), and 6.99% experienced an increase exceeding 50% (ΔCMJ > 50%) (Figure 1C).

**FIG. 2:**
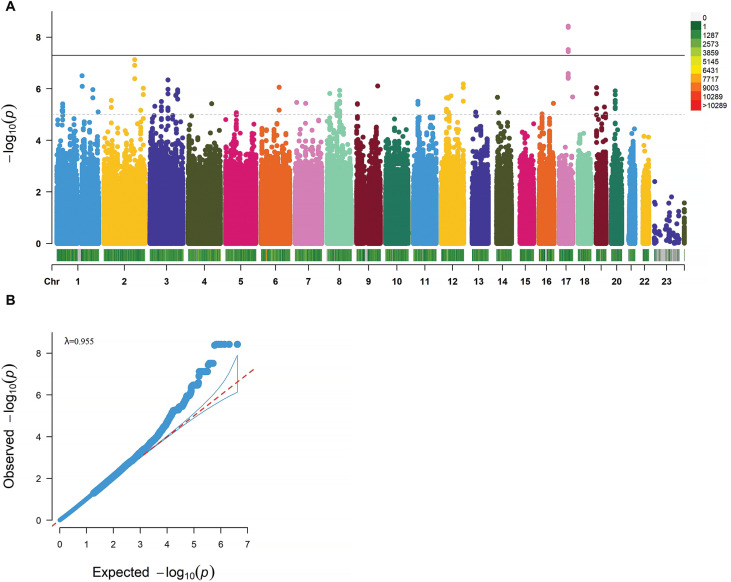
GWAS Analysis of CMJ Height Changes after Resistance Training. A. Manhattan Plot of CMJ Height Change Percentage GWAS Analysis. The x-axis represents chromosomes, with different colors indicating different chromosomes. The color gradient of the bar below the chromosome bar represents the quantity of SNPs on the chromosome, ranging from gray to red to signify an increasing number of SNPs. The y-axis represents -log10(P) values. The dashed line represents a significance threshold of p<1·10-5, and the solid line represents a significance threshold of p<5·10-8. B. Quantile‒Quantile (QQ) Plot and Genomic Inflation Factor λ.

In the study, 4 genome-wide significant SNPs (rs9907859, rs12103525, rs62078596, and rs9893488) located in the PCTP gene (p < 5 × 10^−8^) and 297 SNPs with suggestive significance (p < 1 × 10^−5^) have been identified (Figure 2A). Thirty-eight SNPs were identified as lead SNPs, and the PCTP rs9907859 marker has been selected as one of the 38 lead variants (Table 1).

**TABLE 1:** Lead SNPs Associated with Training Effects on CMJ After Strength Training

SNP	CHR	Position	REF Allele	Minor Allele	MAF	Beta	gwasP	Overlapped Gene	Nearest Upstream Gene	Nearest Downstream Gene
rs9907859	17	53909039	C	G	0.16	56.97	3.75 · 10^−9^	*PCTP*	None	None
rs72894681	2	183507747	A	C	0.05	45.85	7.49 · 10^−8^	None	*PDE1A*	*DNAJC10*
rs7615128	3	107046665	C	A	0.18	38.56	4.57 · 10^−7^	*RP11-446H18.5*	None	None
rs76346437	12	130906425	G	A	0.08	23.41	6.51 · 10^−7^	*RIMBP2*	None	None
rs79611673	9	124537596	G	A	0.01	33.49	7.88 · 10^−7^	*DAB2IP*	None	None
rs3806388	1	150668799	C	A	0.01	28.61	8.03 · 10^−7^	*GOLPH3L*	None	None
rs78489948	6	106915177	T	A	0.07	35.32	8.76 · 10^−7^	None	*Y_RNA*	*AIM1*
rs141592759	19	2499683	C	A	0.07	40.9	9.03 · 10^−7^	None	*GADD45B*	*RNU6-993P*
rs6747425	2	233932621	C	T	0.21	27.09	9.47 · 10^−7^	*INPP5D*	None	None
rs1660385	1	214288379	G	C	0.24	36.51	1.08 · 10^−6^	None	*PROX1*	*SMYD2*
rs1495067	3	164454487	C	T	0.12	32.37	1.11 · 10^−6^	*RP11-71H9.1*	None	None
rs11985065	8	75630494	A	G	0.03	34.15	1.15 · 10^−6^	*RP11-758M4.1*	None	None
rs75438016	8	16835500	A	T	0.01	37.48	1.52 · 10^−6^	None	*RP11-13N12.1*	*FGF20*
rs4378870	20	23699433	A	G	0.32	28.11	1.71 · 10^−6^	None	*CST2P1*	*CST1*
rs701259	3	153618834	G	G	0.09	37.24	1.76 · 10^−6^	*RP11-23D24.2*	None	None
rs79567715	17	79410256	G	A	0.12	22.75	2.08 · 10^−6^	*RP11-1055B8.7*	None	None
rs976221	14	24244706	A	A	0.25	36.25	2.13 · 10^−6^	None	*RP11-388E23.4*	*RN7SKP205*
rs1466789	12	42126705	A	A	0.23	36.62	2.14 · 10^−6^	None	*MTND1P24*	*RP11-630C16.1*
rs77973780	12	31140920	C	T	0.02	30.33	2.24 · 10^−6^	*TSPAN11*	None	None
rs2167978	2	44353077	A	T	0.12	31.17	2.83 · 10^−6^	None	*AC019129.1*	*RNU6-566P*
rs113021171	11	27048503	G	T	0.15	27.66	3.07 · 10^−6^	None	*FIBIN*	*BBOX1*
rs118191183	3	68243115	G	T	0.02	28.24	3.10 · 10^−6^	*FAM19A1*	None	None
rs75380147	7	9985223	T	C	0.05	33.53	3.40 · 10^−6^	None	*GS1-69O6.1*	*AC006373.1*
rs34337282	16	85599995	G	A	0.14	38.87	3.66 · 10^−6^	None	*AC092377.1*	*RP11-118F19.1*
rs61050775	4	138564605	A	G	0.15	31.66	3.79 · 10^−6^	None	*RP11-714L20.1*	*RP13-884E18.2*
rs12343431	9	4759946	C	A	0.16	34.24	3.81 · 10^−6^	None	*AK3*	*RP11-307I14.4*
rs35658380	1	34845898	C	T	0.24	21.62	3.87 · 10^−6^	None	*RP4-657M3.2*	*MIR552*
rs7834774	8	59631013	G	A	0.14	26.72	3.90 · 10^−6^	None	*snoU13*	*TOX*
rs79531236	3	29413111	A	G	0.03	33.92	4.80 · 10^−6^	*RBMS3*	None	None
rs2303690	19	48525507	G	G	0.31	30.22	5.07 · 10^−6^	*ELSPBP1*	None	None
rs72613753	2	218460125	A	G	0.06	18.49	6.84 · 10^−6^	*DIRC3*	None	None
rs373545195	1	31720006	T	C	0.01	39.44	7.00 · 10^−6^	None	*NKAIN1*	*SNRNP40*
rs80010151	1	244954099	C	T	0.05	32.9	7.91 · 10^−6^	None	*DESI2*	*COX20*
rs80068293	13	40144130	A	G	0.03	29.6	8.07 · 10^−6^	*LHFP*	None	None
rs59224847	5	63480499	G	C	0.18	30.38	8.47 · 10^−6^	*RNF180*	None	None
rs2284654	14	31346510	C	A	0.08	29.21	8.48 · 10^−6^	*COCH*	None	None
rs4265793	16	19028549	T	T	0.11	32.7	9.41 · 10^−6^	*TMC7*	None	None
rs61448344	19	55587668	C	T	0.16	22.93	9.55 · 10^−6^	*EPS8L1*	None	None

The genomic inflation factor (λ) was calculated to be 0.955 (λ≈1), indicating that the p values were not skewed by population stratification and that false positive results were not present (Figure 2B).

A stepwise linear regression analysis revealed that the initial CMJ value, sex, PGS, muscle strength, and body mass were significant predictors of training effects on power, resulting in a model explanatory power of 62.6% (R^2^ = 0.626) In this regard, females are coded as 0 and males as 1. A positive B or beta value indicates that the predictor is positively associated with CMJ increase, while a negative value indicates a negative association. (Table 2).

**TABLE 2:** Prediction Model for Training Effects on CMJ Using “PGS-Phenotypic Indicators”

Coefficient	Unstandardized Coefficients		Standardized Coefficients Beta	Sig.	R^2^	Adjusted R^2^
	B	Std. Erro				
Constant	95.55	7.913		< 0.001		
Initial CMJ height	-3.029	0.189	-1.133	< 0.001	0.138	0.133
Sex	38.915	3.617	0.847	< 0.001	0.299	0.298
PGS	3.758	0.517	0.35	< 0.001	0.13	0.129
Initial 1RM squat value	-0.53	0.111	-0.281	< 0.001	0.031	0.029
Body mass	0.205	0.056	0.276	< 0.001	0.028	0.027

**FIG. 3:**
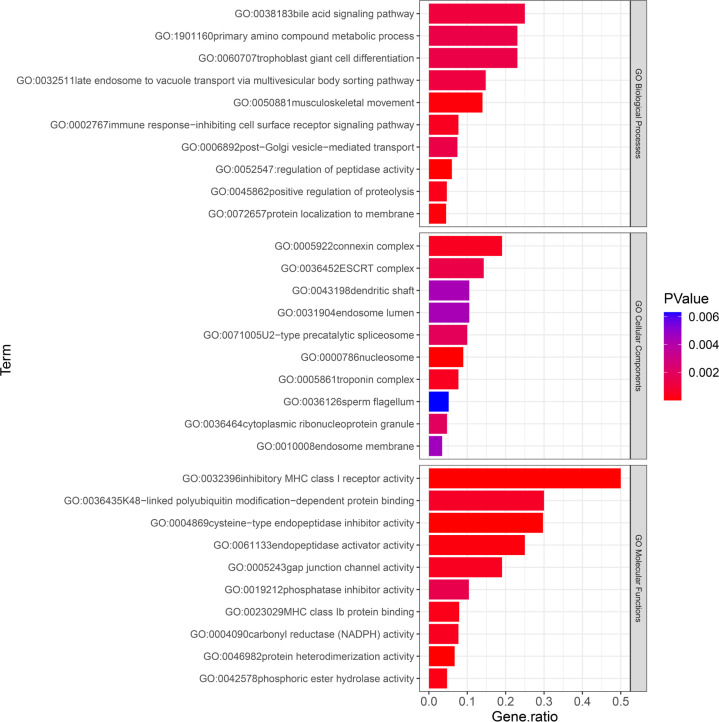
. GO enriched terms. Listed in order of p-values, showing the top 10 terms for Biological Processes (BP), Cellular Components (CC), and Molecular Functions (MF). The p-values are color-coded, ranging from blue to red, indicating decreasing values.

The results of the GO analysis revealed that the mapping genes of SNPs associated with the training effect of power after resistance training were primarily enriched in 29 biological process terms, 10 cellular components, and 16 molecular functions. Among these, the “musculoskeletal movement” biological process term may be related to power (Figure 3).

**FIG. 4:**
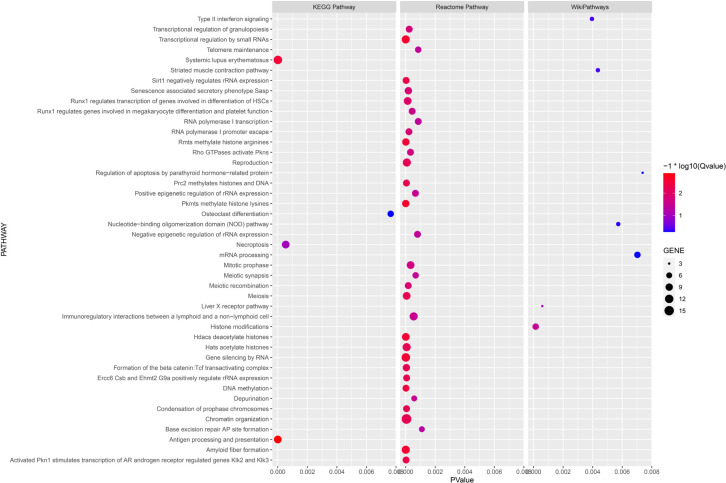
KEGG enriched terms. The size of the bubbles represents the number of genes enriched in that pathway, while the color indicates the magnitude of -log10(Q-values) after p-value correction, transitioning from blue to red as the p-values decrease.

Enrichment analysis using the KEGG Pathway, Reactome Pathway, and WikiPathways databases yielded 4, 33, and 7 pathways, respectively (Figure 4). Of particular interest, the “Striated muscle contraction” pathway within the WikiPathways signaling pathway analysis may have relevance to the training effect of power after resistance training.

## DISCUSSION

This study presents an assessment of individual differences in the training effects on power subsequent to resistance training. Notably, this is the first study to identify training-sensitive genetic markers at the whole-genome level, identifying a total of 38 lead SNPs. The predictive model developed for the training effects on power, which combines polygenic score (PGS) and phenotypic indicators, achieved a notable explanatory power of 62.6%. Specifically, PGS accounted for 13.0% of the explanatory power, while the phenotypic indicators contributed significantly to 49.6%. Furthermore, through bioinformatics analysis, the lead SNPs were found to potentially participate in key biological processes associated with musculoskeletal movement, as well as the striated muscle contraction pathway.

In this study, we observed an overall improvement in lower limb power after regular resistance training; however, individual differences in the training effects were apparent. Previous studies have demonstrated the positive impact of resistance training on increasing the CMJ height among athletes [17]. Untrained participants also exhibited a significant increase in CMJ height after an 8-week resistance training program (from 23.90 cm to 26.90 cm) [18]. Furthermore, even in older adults, a 12-week moderate-intensity resistance training program led to a notable 31% enhancement in CMJ vertical jump height [19]. These studies primarily focused on the intervention effects of resistance training on the population as a whole and did not delve into individual differences in response to resistance training. In reality, variations in improving 1RM muscle strength, muscle volume, muscle cross-sectional area, and other training outcomes are commonly observed among individuals undergoing resistance training [1, 20, 21]. Nonetheless, few studies have explored individual differences in CMJ height improvement through resistance training. In our study, the average increase in CMJ height among participants was 16.53%, with individual responses ranging from -35.90% to 125.71%. These findings indicate that not all participants exhibit favorable adaptations to resistance training.

Genetics and participant backgrounds are likely to be the main factors influencing individual differences in response to resistance training. Association studies have identified numerous genetic variations associated with training response and exercise-related traits. Carriers of PPARGC1A rs8192678 Gly/Gly, PPARD rs1053049 TT, PPARD rs2267668 AA, and PPARG rs1801282 Ala alleles exhibit the best response to aerobic training [3]. MCT1 rs1049434 has also been reported to be associated with athletic performance and physiological phenotypes [22]. Undoubtedly, there may be additional genetic variations associated with the response to different resistance training modalities, warranting further exploration at the genomewide level. In this study, we identified 38 lead SNPs that exhibited significant associations with the percentage change in CMJ height following resistance training. It is important to note that not all SNPs associated with phenotype traits have equal effects. Lead SNPs, which exert a dominant role and demonstrate the strongest correlation with the phenotype, play a crucial role in this context [23]. Although limited, several studies have already examined the association between genetic markers and baseline CMJ height. For instance, genes such as *ACE* and *ACTN3* are considered to be related to muscle phenotype. However, in Polish athletes, no association was found between *ACE* and *ACTN3* genotypes and CMJ height [24]. In Italian football players, *ACE* and *ACTN3* genotypes were found to have no significant association with CMJ height but showed associations with SJ (squat jump). Moreover, when combined with the level of competition, these genotypes explained 23.92% of the individual differences [25]. The D allele of the *ACE* gene has been observed to confer an advantage for high CMJ height in male and female sprint athletes, as well as in female endurance athletes [26]. However, research specifically focusing on genetic markers related to CMJ height responsiveness to resistance training is currently lacking.

The lead SNPs identified in this study are primarily located in intronic regions, UTR regions, and intergenic regions. For example, the most significant lead SNP, rs9907859, is situated in an intronic region of the Phosphatidylcholine Transfer Protein *(PCTP)* gene. *PCTP* plays a significant role in brown adipose tissue (BAT). *PCTP* (-/-) mice lacking phosphatidylcholine transfer protein (Pctp) tend to utilize fatty acids for oxidative phosphorylation, suggesting a potential regulatory role of Pctp in the pathway of fatty acyl-CoA entry into mitochondria [27]. Hepatic deletion of *PCTP* has been shown to reduce adipose tissue mass and lower levels of triglycerides and phospholipids in skeletal muscle [28]. While this study suggests a potential impact of *PCTP* on skeletal muscle energy metabolism, research concerning rs9907859 remains limited, necessitating further investigation in the future. These findings imply that *PCTP* may have an impact on skeletal muscle energy metabolism. However, research regarding rs9907859 remains scarce, necessitating further investigation in the future. Another SNP, rs79531236, is located in an intronic region of the *RBMS3* gene, which is believed to play a role in muscle cell proliferation and gastrointestinal motility. It has also been associated with gastrointestinal abnormalities in patients with *FOXP1* syndrome [29]. Some SNPs are located on lincRNAs, referring to long noncoding RNAs with a length greater than 200 nucleotides. LncRNAs fulfill various roles, including epigenetic regulation, modulation of chromatin structure, transcriptional control, regulation of mRNA stability, translation, and posttranslational modifications [30]. The SNP rs7615128 is located on the lncRNA *RP11-446H18.5,* and it may have some impact on the functionality of the lncRNA. Additionally, there are other SNPs, such as rs72894681, rs78489948, and rs141592759, identified in this study, which are situated between genes. SNPs located between genes are potentially in proximity to transcription factor-binding sites, enhancers, promoters, or other regulatory elements that can influence the expression and regulation of both genes. However, the specific role of these SNPs in regulating gene expression and their association with muscle strength phenotypes remains unknown within the context of this study.

A regression model was constructed using PGS and phenotype indicators (initial CMJ value, sex, age, and total body muscle mass) to account for 62.6% (R^2^ = 0.626) of the variance in CMJ height following resistance training. Previous studies have employed Spearman’s correlations to assess the relationship between genotype score and the percentage change in CMJ height, with the genotype score explaining 14–32% of the variance in CMJ height [31], which aligns with the findings of this study. These findings suggest that genetic markers can partially elucidate the effectiveness of power training after resistance training. Genetic factors, as represented by the PGS utilized in this study, accounted for 13.0% of the variation observed, while the phenotype indicators contributed to 49.6% of the variation in explaining the effectiveness of power training post-strength training. This comparison suggests that the genetic factors captured by the PGS constituted a proportion of the overall genetic influence on the training effect. However, it is important to note that the PGS constructed in our study is based on a subset of common SNPs and does not encompass the entirety of genetic variability. We acknowledge that there are likely other genetic elements such as rarer variants, structural variations, and unmeasured genetic factors that could contribute to the overall genetic component influencing training outcomes. Thus, while our findings highlight a partial influence of the included genetic factors, we recognize the limitations in capturing the complete spectrum of genetic determinants affecting training effects. Some SNPs in genes like *ACE, ACTN3, PPARs,* and *MCT1,* which were found in previous studies to be related to training outcomes [3, 22, 24], were not included in the PGS constructed in this study. This omission might be attributed to the focus of this study on the CMJ height indicator, which differs from previous research. It also suggests that different genetic determinants may underlie the training effects of different indicators. The negative correlation between the initial CMJ value and the training effect on CMJ height suggests that individuals with lower initial power are more likely to experience improvements through regular resistance training.

Sex is another factor influencing the effectiveness of power training. In our study, we observed variations in the extent of changes between males (-20.14% to 125.71%) and females (-35.90% to 76.85%). There are discrepancies in neuromuscular recovery after resistance training between sexes, with females displaying a more pronounced decrease in CMJ height than males [32]. This indicates that sex differences exist in the response of CMJ height to the stimulus of resistance training. Another study also identified sex and age disparities in muscle fiber types (I, IIa, and IIx) and muscle crosssectional area following long-term resistance training [33]. Since muscle fiber type is a determinant of power, sex differences in adaptation to resistance training may contribute to the influence of sex and age on power prediction. We discovered that lower limb muscle strength (1RM) holds predictive value for power. Muscle strength serves as the foundation for generating power, and participants with superior lower limb strength can achieve greater enhancements in power after resistance training. Although body mass may not directly impact CMJ height, it exhibits a positive correlation with CMJ peak power [34]. Muscles serve as the direct source of power, and augmenting relative maximum strength can enhance performance in lower limb power movements [35]. However, in the predictive model, muscle mass was not included in the regression analysis of individual differences in power. This may suggest that the effectiveness of power training relies less on muscle mass.

The bioinformatics analysis conducted in this study revealed that the SNPs linked to the training effects of power are involved in various biological processes, molecular functions, and cellular components, extending beyond skeletal muscle or muscle strength. For instance, these SNPs may be implicated in the biological process of positive regulation of proteolysis. Positive regulation of proteolysis plays a crucial role in multiple biological processes, including cellular homeostasis, protein metabolism, and signaling pathway regulation. Moreover, the “antigen processing and presentation” pathway in the KEGG database is a vital process in the immune system, facilitating the recognition and response to various foreign antigens [36]. Additionally, the “PKMT methylate histone lysines” pathway in the Reactome Pathway has the potential to influence chromatin structure and transcriptional activity of genes, thereby regulating cellular function and phenotype. It plays a critical role in biological processes such as development, cell differentiation, and disease occurrence [37].

Research directly connecting SNPs to the power phenotype is still limited. Through bioinformatics analysis, this study identified SNPs (rs976221, rs61448344, rs6747425, rs141592759) associated with the effectiveness of power training, suggesting their potential involvement in the biological process of musculoskeletal movement. The analysis revealed four SNPs (rs976221, rs61448344, rs6747425, rs141592759) and six mapping genes *(MYH7, TNNI3, TNNT1, GIGYF2, JSRP1, PVALEF)* that are closely associated with the identified SNPs. These genes have been shown to contribute to various growth, development, or physiological processes of skeletal muscle. For instance, the *MYH7* gene, one of the genes in the human genome encoding the beta-myosin heavy chain, encodes slow/ cardiac MyHC (*MyHC* I), which is expressed in slow, oxidative, type 1 fibers of skeletal muscle. It plays a role in multiple biological processes related to muscle contraction, such as regulating slow-twitch skeletal muscle fiber contraction and the force of skeletal muscle contraction [38]. Variations in the *MYH7* gene have also been confirmed to be associated with skeletal muscle diseases [39].

In this study, no muscle-specific signaling pathways were identified in the KEGG Pathway and Reactome Pathway databases. However, the Wikipathway database revealed the potential influence of SNPs on the striated muscle contraction pathway, which could be related to the effectiveness of power training. This pathway involves four mapped genes: *ACTG1, MYH6, TNNI3,* and *TNNT1,* which are closely associated with skeletal and cardiac muscles, playing crucial roles in the structure and function of skeletal muscle. For instance, the *ACTG1* gene encodes the γ-actin protein, which is a component of actin in skeletal muscle cells. Actin binds with myosin and participates in muscle contraction and movement. Studies have shown that in a mouse model with *ACTG1* deletion (Actg1-msKO), muscle development remains normal, but Actg1-msKO mice display significant muscle weakness accompanied by a progressive pattern of muscle fiber degeneration/regeneration [40].

GWASs have identified a genetic variation (rs6565586) in the *ACTG1* gene that is associated with the status of power-oriented athletes [41]. Additionally, *ACTG1* rs6565586 has been confirmed to be related to the grip strength [42]. *MYH6* and *TNNT1* are crucial for maintaining normal skeletal muscle function, and variations in these genes may lead to skeletal muscle diseases [43, 44]. In summary, these genes play essential roles in regulating skeletal muscle structure and function. Mutations or functional abnormalities in these genes may contribute to the occurrence of skeletal muscle diseases such as myopathies and muscular atrophy. They may also be involved in determining the power phenotype, although further research is necessary to confirm this.

### Limitations

When reviewing our study outcomes, we acknowledge several limitations in our research. Firstly, we utilized a relatively lenient significance threshold (p-value of 1 × 10^−5^) to identify associations between genes and training effects. This choice was based on the relatively modest sample size of our study cohort, the nature of the genetic markers analyzed, and the necessity to strike a balance between reducing false positives and identifying potential related genetic variations [15, 45]. This selection may have excluded potential correlations detectable under stricter thresholds, thus warranting recognition that certain crucial genetic variations might have been overlooked. Additionally, our study did not comprehensively assess the random variation in training effects among participants. For instance, we did not quantify the variability in the primary phenotype within the control group, nor did we assess the reliability (repeatability) of training effects (primary phenotype) within the training group. Evaluating these aspects would have contributed to a more comprehensive understanding of the variability and credibility of training effects. Finally, despite our efforts to screen genes related to training effects from 4 million SNPs, it is possible that other SNPs, rare variants, or structural variations were not included. This suggests that our study outcomes might not encompass all potential genetic factors contributing to training effects. We acknowledge the importance of replication studies in validating our GWAS findings, particularly those concerning the 37 suggestively significant lead SNPs, before their practical application in predicting CMJ increase. Overall, while our study provides valuable insights, these limitations remind us to interpret our results cautiously. Furthermore, they suggest directions for future research to comprehensively understand the influence of genes on training effects.

## CONCLUSIONS

After 12 weeks of resistance training, individual variations were observed in the percentage change of CMJ. The predictive model developed using PGS and phenotypic indicators explained 62.6% of the variance in the percentage change of CMJ. Specifically, the PGS accounted for 13.0% of the variance, whereas phenotypic indicators contributed 49.6%. It is suggested that SNPs associated with CMJ variation may impact the training effects on CMJ changes through the striated muscle contraction pathway.
